# Neurological Associations Among COVID-19 Patients: A Systematic Review and Meta-Analysis

**DOI:** 10.1007/s44229-022-00010-1

**Published:** 2022-06-10

**Authors:** Nashwa Radwan, Nagla Mahmoud, Abdullah Alkattan, Amal Alfaifi, Khaled Alabdulkareem

**Affiliations:** 1 Assisting Deputyship for Primary Health Care, Ministry of Health, Riyadh, Kingdom of Saudi Arabia; 2 Department of Public Health and Community Medicine, Faculty of Medicine, Tanta University, Tanta, Egypt; 3 Department of Research and Development, General Directorate of School Health, Ministry of Health, Riyadh, Kingdom of Saudi Arabia; 4 Department of Biomedical Sciences, College of Veterinary Medicine, King Faisal University, Hofuf, Al-Ahsa Kingdom of Saudi Arabia; 5 Department of Family Medicine, College of Medicine, Al-Imam Mohammad Bin Saud Islamic University, Riyadh, Kingdom of Saudi Arabia

**Keywords:** Neurological complications, COVID-19, Risk factors, Cerebrovascular complications

## Abstract

**Background:**

The global threat of COVID-19 caused by the SARS-CoV-2 virus has reached a high level and the outbreak has been declared as a pandemic. This disease affects different organs and systems including the central nervous system. In this study, we aimed to clarify the development of neurological complications in patients with COVID-19 and the factors associated with these conditions.

**Methodology:**

Two authors independently searched the Cochrane, Trip, EMBASE, and Google Scholar databases from January 2020 to February 2021. The literature search included studies written in English and related to neurological complications in COVID-19 patients. Then, the two authors independently determined the characteristics and risk of bias of the included studies. Finally, we analyzed the data using odds ratios (ORs) or mean differences (MDs) and 95% confidence intervals (CIs).

**Results:**

This review involved 4401 patients with COVID-19 from six observational studies. Overall, low to moderate heterogeneity was recorded among the included studies. A high risk of bias was not detected in any of the domains studied, although there were some low risks of bias and heterogeneity. Of the included patients, 8.24% developed neurological manifestations, including delirium (84.3%), myalgia (44.8%), headache (37.7%), encephalopathy (31.8%), dizziness (29.7%), dysgeusia (15.9%), anosmia (11.45), acute ischemic stroke (4.6%), cerebrovascular disease (1.78%), and intracerebral hemorrhage (0.5%). The severity of COVID-19 and the association of underlying comorbidity (predominantly hypertension) increased the risk of neurological complications among COVID-19 patients by fourfold (OR 4.30, CI 2.54–7.29 and OR 4.01, CI 1.05–15.36, respectively). Patients with heart diseases, diabetes, and dyslipidemia had a twofold higher risk of developing neurological complications (OR 2.53, CI 1.01–6.33; OR 2.31, CI 1.15–4.65; and OR 2.13, CI 1.52–3.00, respectively).

**Conclusion:**

Our analysis indicated that neurological complications were uncommon in patients with COVID-19. Age, male sex, smoking, the severity of disease, and underlying comorbidity, including hypertension, heart disease, diabetes, and dyslipidemia, were identified as significant risk factors for neurological complications in COVID-19 patients.

## Background

Since the discovery of SARS-CoV-2 until August 2021, 140 million people have been infected with more than three million deaths all over the world [1–4]. The global threat of COVID-19 caused by the SARS-CoV-2 virus has reached a high level and the outbreak has been declared by the World Health Organization (WHO) as a pandemic [5]. Upper and lower respiratory symptoms are the most common presentation of COVID-19; however, this disease can affect any of the organs and the central nervous system (CNS). These neurological complications include dizziness, headache, impaired consciousness, encephalopathy, stroke, and delirious manifestations [6]. Brain injury and neurological symptoms might occur via invasion of the neural cell membrane by angiotensin converting enzyme-2 (ACE2). Affects in the CNS could also be caused by viral infection occurring via axonal transport of the virus by peripheral nerves into the brain [6]. An alternative theory is that the CNS becomes inflamed due to nonspecific complications of the systemic disorder [7]. Previous studies showed that COVID-19 patients with chronic diseases are more likely to develop neurological complications [8–10]. However, the specific mechanisms responsible for how this virus affects the CNS have yet to be elucidated. Therefore, in this review, we aimed to investigate the development of neurological complications in COVID-19 patients and the mechanisms that occur in these events.

## Methodology

### Inclusion/Exclusion Criteria

This review involved publications that were written in English and investigated neurological complications in patients with COVID-19 from 2019 until now. We excluded studies that did not confirm COVID-19 infection and studies that involved neurological patients who were subsequently infected with COVID-19. We also excluded case reports and case series that investigated neurological associations with COVID-19 due to the absence of a comparison group.

### Search Methods

Two authors independently searched the Cochrane, Trip, EMBASE, and Google Scholar databases from 2019 until now. In addition, we reviewed the reference lists of the selected articles for further studies. We used the following terms in the search: “COVID-19,” OR “novel corona,” OR “severe pneumonia,” OR “patients infected with corona and neurological manifestation,” OR “neurological complications,” OR “cerebrovascular complications,” OR “encephalopathy,” OR “encephalitis,” OR “delirium,” OR “ischemic stroke,” OR “brain magnetic resonance imaging (MRI).”

### Outcome Measures

The outcome measures were the development of neurological complications in COVID-19 patients including CNS manifestations (e.g., headache, encephalopathy, and stroke), peripheral nervous system (PNS) impairment (e.g., dysfunction of taste, dysfunction of smell, neuropathy), and skeletal muscle manifestations (e.g., myalgia).

The confirmation of COVID-19 disease was based on WHO guidelines [11]. Patients were classified as having severe COVID-19 based on the American Thoracic Society Infectious Disease guidelines [12]. The level of consciousness was assessed using the Richmond Agitation-Sedation Scale [13]. The assessment of neurological complications was confirmed by (1) the onset of neurological manifestations within 6 weeks of acute COVID-19 infection, and (2) either the detection of SARS-CoV-2 RNA in a given sample or detection of the antibody for the infection with no evidence of other commonly associated causes [14].

### Data Collection and Extraction

The abstracts of screened articles were reviewed independently by two authors (NR, NM). Articles fitting the inclusion criteria were acquired and their characteristics were determined independently. These characteristics included the study setting, duration, and design, participant age and sex, and outcome measures. Any disagreements were resolved by discussion.

### Assessment of the Risk of Bias

Two authors (NR, NM) independently judged the risk of bias for the included studies. The risk of bias was graded as high, low, or unclear, as recommended by The Newcastle Ottawa Scale for nonrandomized studies [15].

### Assessment of the Quality of Evidence

The quality of evidence for each outcome measure was judged as high, moderate, low, or very low according to the GRADE approach (Grading of Recommendations Assessment, Development, and Evaluation) [16].

### Measures of Treatment Effect

A random effect model was created in Collaboration C. Review manager (RevMan) version 5.3. The Nordic Cochrane Centre: Copenhagen, Denmark 2014 software [17] and utilized for data analysis. Odds ratios (ORs) or mean differences (MDs) with 95% confidence intervals (CIs) were utilized for dichotomous and continuous data, respectively.

### Dealing with Heterogeneity

The *I*^2^ test was used to test the heterogeneity of the studies included in our analysis [18].

## Results

### Results of Search

**Fig. 1:**
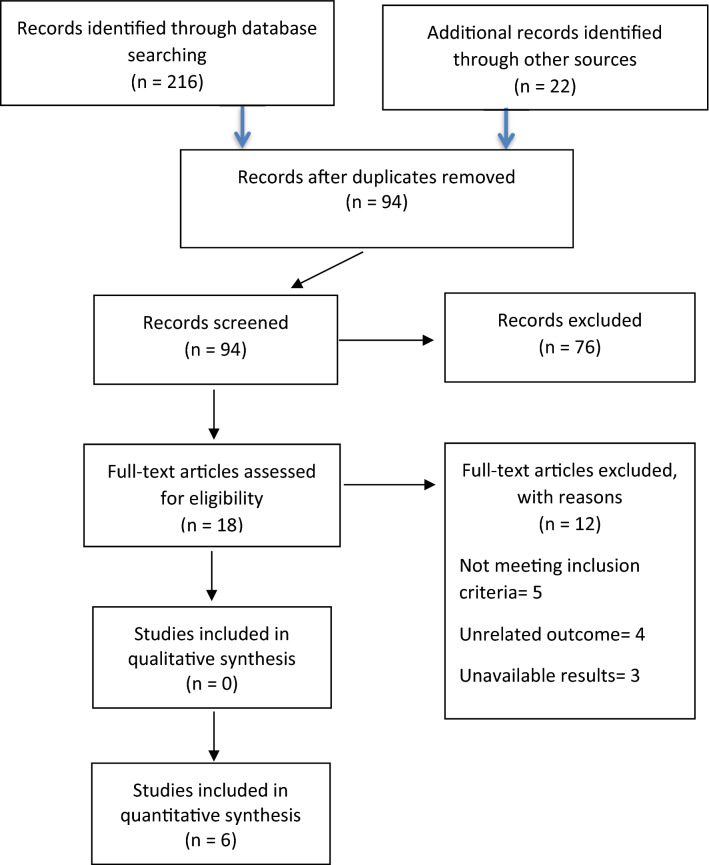
PRISMA flow diagram describing the search process and the studies included

Two hundred and sixteen potentially relevant articles were identified; 94 of these remained after the exclusion of duplicates. The abstracts of these articles were subsequently appraised with regards to the inclusion criteria. Eighteen full text articles were evaluated for eligibility; six of these met the inclusion criteria. A PRISMA flow diagram is shown in Fig. 1 and provides further details of the search method used and the studies included.

### Included Studies

The review included six studies [19–24] (four retrospective, one prospective, and one retrospective cohort studies). Two authors independently extracted key characteristics from the selected studies, including study title, journal, study design, duration, setting, aim, participant age, sex, number, and outcome measures (Table 1).

**Table 1:** Characteristics of the included studies

Author	Title	Setting/duration	Design	Participants	Aim	Outcome
Li et al. [19], 2020	Acute cerebrovascular disease following COVID-19: a single center, retrospective, observational study	Union Hospital of Huazhong University of Science and Technology between 16 January and 19 February 2020	Single-center retrospective study	219 patients with COVID-19	To describe the clinical characteristics, laboratory features, and outcomes of cardiovascular disease complicating SARS-CoV-2 infection	4.6% patients developed acute ischemic stroke and 0.5% had intracerebral hemorrhage
Liotta et al. [20], 2020	Frequent neurologic manifestations and encephalopathy-associated morbidity in Covid-19 patients	Hospital network in Chicago, IL, between 5 March and 6 April 2020	Retrospective study	509 consecutive patients admitted with confirmed COVID-19 within a hospital network in Chicago, IL	To characterize the neurological manifestations, their risk factors, and associated outcomes in hospitalized patients with COVID-19	Neurological manifestations were present at the onset of COVID-19 in 215 (42.2%)
Merkler et al. [21], 2020	Risk of ischemic stroke in patients with coronavirus disease 2019 (COVID-19) vs patients with influenza	Conducted at two academic hospitals in New York City, 4 March 2020 to 2 May 2020	Retrospective cohort study	2132 patients with emergency department visits or hospitalizations with COVID-19	To compare the rate of ischemic stroke between patients with COVID-19 and patients with influenza	31 patients (1.5%; 95% confidence interval [CI] 1.0%–2.1%) had an acute ischemic stroke
Kandemirli et al. [22], 2020	Brain MRI findings in patients in the intensive care unit with COVID-19 infection	Eight hospitals (two university hospitals and six university-affiliated hospitals between 1 March and 18 April 2020	Retrospective study	749 inpatients with COVID-19 infection	To describe brain MRI findings in the evaluation of patients in the intensive care unit (ICU) with COVID-19 pneumonia	Fifty of the 235 ICU patients (21%; 95% CI 16%–27%) developed neurological symptoms
Guan et al. [23], 2020	Comorbidity and its impact on 1590 patients with COVID-19 in China: a nationwide analysis	575 hospitals in 31 provinces/autonomous regions/provincial municipalities across mainland China between 11 December 2019 and 31 January 2020	Retrospective case study	1590 laboratory-confirmed COVID-19 hospitalized patients	To evaluate the risk of serious adverse outcomes in patients with COVID-19	Severe cases accounted for 16.0%. (25.1%) reported having at least one comorbidity
Helms et al. [24]	Delirium and encephalopathy in severe COVID-19: a cohort analysis of ICU patients	Two French ICUs of Strasbourg University Hospital between 3 March and 5 May 2020	Cohort study	140 patients referred for COVID-19	To describe delirium and neurological symptoms of COVID-19 in ICU patients	(84.3%) developed delirium

### Trial Participants

The review included 4401 COVID-19 patients; 2496 were male. The mean age of the patients reported by Li et al. [19], Guan et al. [23] and Liotta et al. [20] were 75.7 ± 10.8, 48.9 ± 9.6, and 58.5 ± 16.9 years, respectively. The median age of the patients was 63 years (range 34–87 years), 62 years (range 52–70 years) and 69 years (range 66–78 years) in the studies reported by Kandemirli et al. [22], Helms et al. [24], and Merkler et al. [21], respectively.

### Risk of Bias Among the Included Studies

**Fig. 2:**
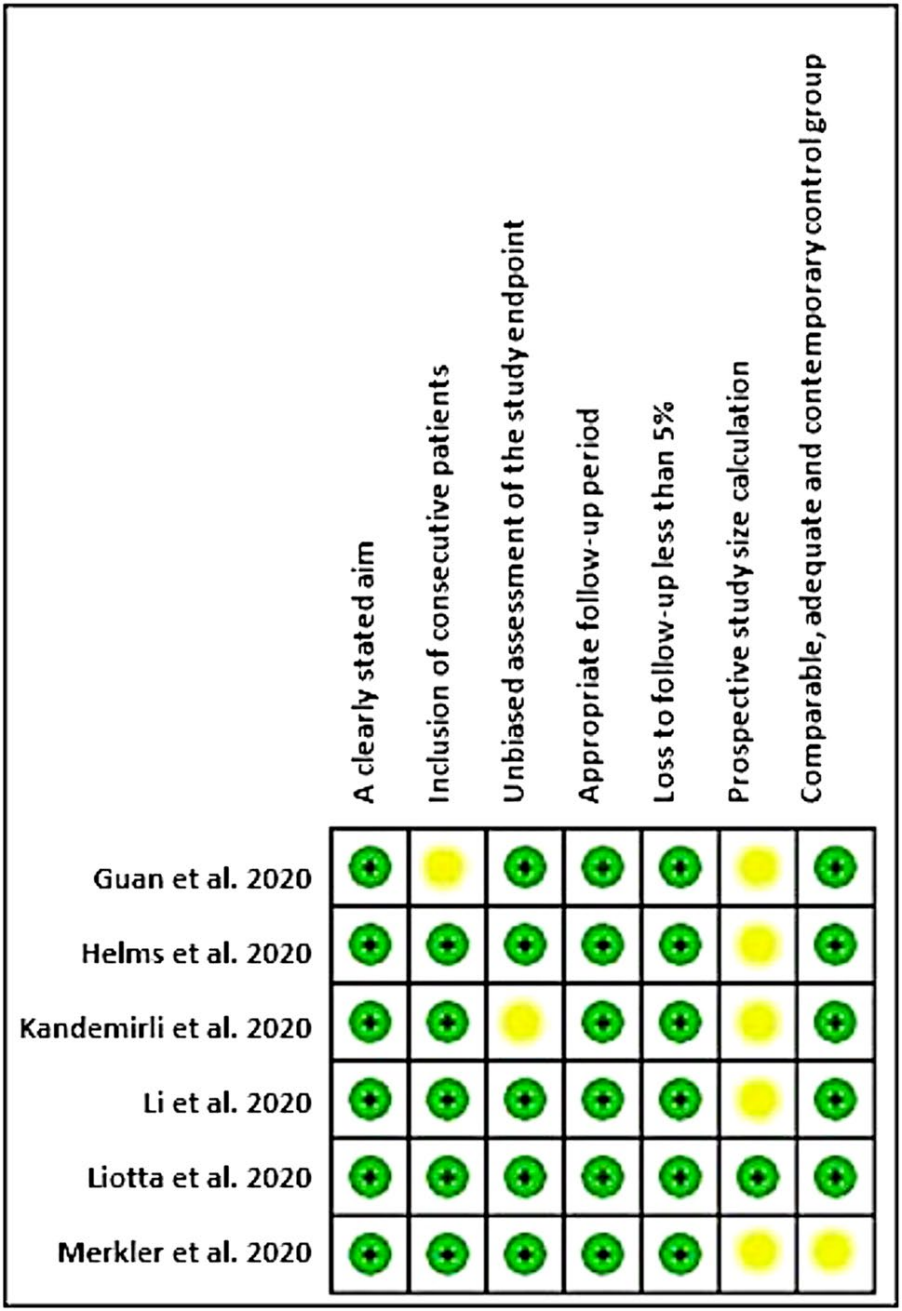
Risk of bias among the studies included in this study, according to the authors' judgements

Overall, the included studies recorded a low risk of bias for most of the studied domains and no high risk of bias was recorded for any domain. The risk of bias was unclear for the “prospective calculation of the study size” domain in the studies reported by Guan et al. [23], Helms et al. [24], Kandemirli et al. [22] and Merkler et al [21]. In addition, an unclear risk of bias was recorded for the “inclusion of consecutive patients” domain reported by Guan et al [23], the “unbiased assessment of study endpoint” domain reported by Kandemirili et al. [22], and the “comparable, adequate and contemporary control” domain reported by Merkler et al. [21] (Fig. 2).

### Outcome Measures

Of the 4401 subjects included in this analysis, 364 (8.27%) had neurological manifestations. In the study reported by Li et al. [19], 10 out of 219 patients (4.6%) developed acute ischemic stroke and 1 (0.5%) suffered from an intracerebral hemorrhage. In Helms et al [24], 118 out of 140 (84.3%) patients developed delirium with cognition disturbances. In Kandemirli et al [22], 118 out of 235 (50%) patients developed neurological symptoms; 44% of those who underwent brain magnetic resonance image showed acute findings. These findings included subcortical and deep white matter signal intensity abnormalities in the frontal lobe in four patients, the parietal lobe in three patients, the occipital lobe in four patients, the temporal lobe in one patient, the insular cortex in three patients, and the cingulate gyrus in three patients. In Liotta et al. [19], 215 out of 509 (42.2%) patients developed neurological manifestations, including myalgia (44.8%), headache (37.7%), encephalopathy (31.8%), dizziness (29.7%), dysgeusia (15.9%), and anosmia (11.4%); strokes, movement disorders, motor and sensory deficits, ataxia, and seizures accounted for 0.2% to 1.4% of cases. In the study reported by Guan et al. [23], 30 out of 1690 (1.78%) developed cerebrovascular disease, including multiple lobe infiltration, delirium, or the loss of consciousness. In Merkler et al. [21], 31 (1.6%) of the 1916 patients had an acute ischemic stroke.

**Fig. 3:**
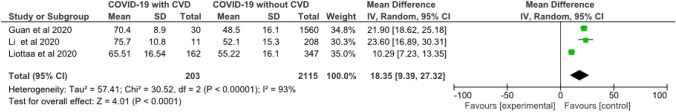
Forest plot of the mean age of COVID-19 cases with and without neurological manifestations

Figure 3 shows a forest plot of the mean age across the included studies. Analysis included three studies involving 2318 participants. Old age was identified as a statistically significant risk factor for developing neurological complications in COVID-19 patients (MD 18.35, CI 9.39–27.32). Significant heterogeneity was also noted (*I*^2^ = 93%, *P* < 0.00001).

**Fig. 4:**
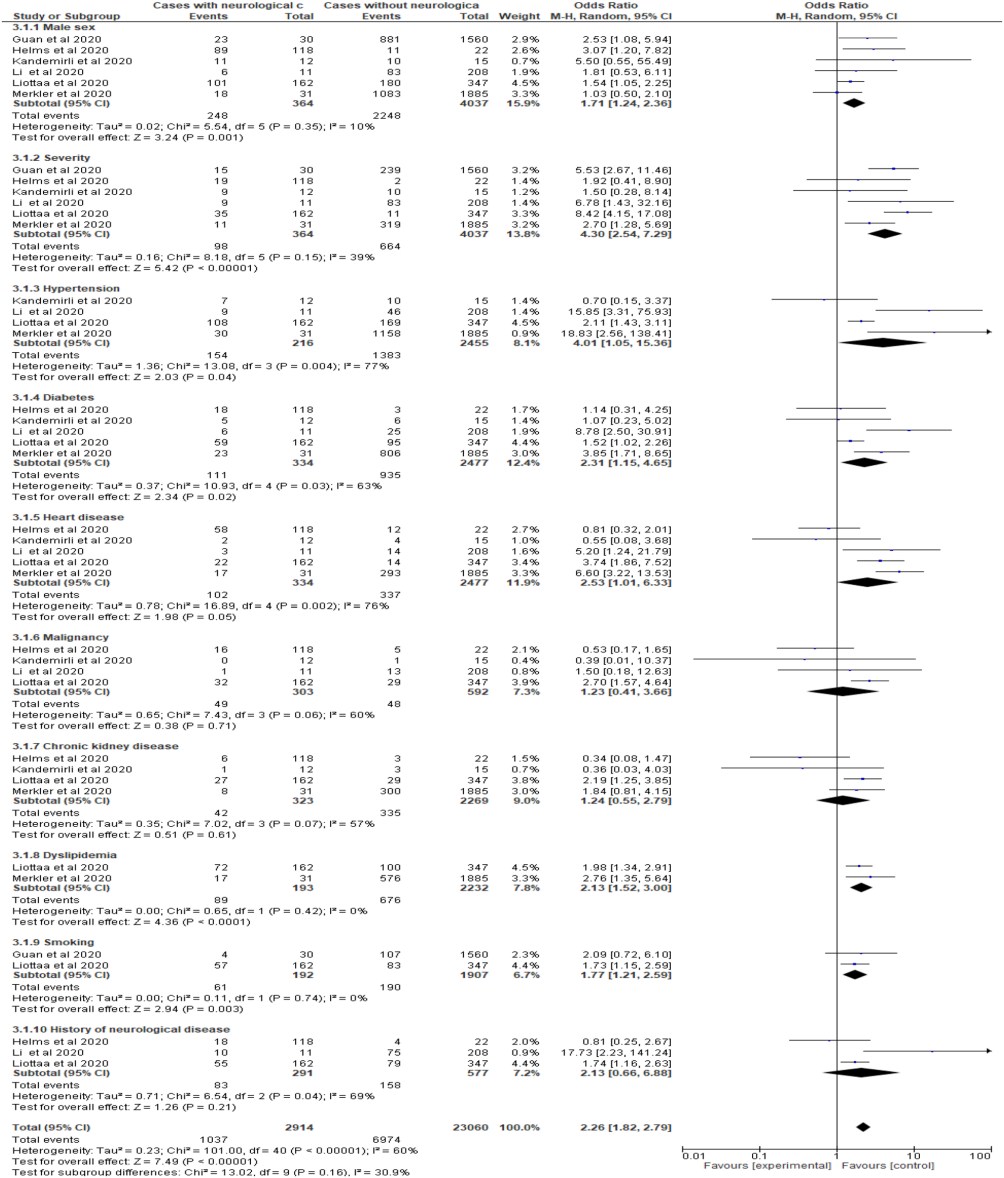
Forest plot of the risk factors for developing neurological complications in COVID-19 patients

**Fig. 5:**
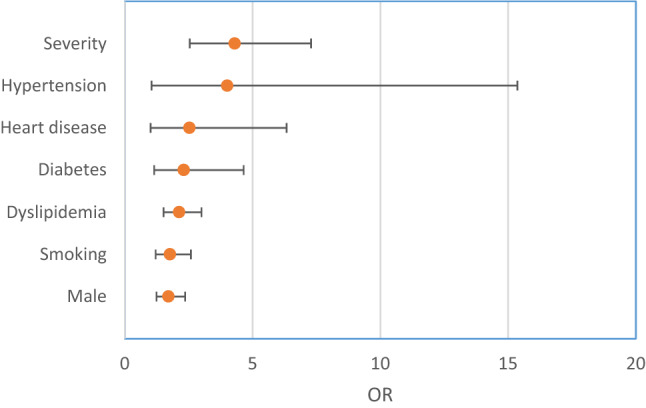
Forest plot of the pooled estimate of significant risk factors for neurological associations in COVID-19 patients

Figure 4 shows a forest plot of factors associated with the development of neurological complications in COVID-19 patients. The significant risk factors for neurological associations were as follows: severity of COVID-19 (OR 4.30, CI 2.54–7.29, *I*^2^ = 39%), hypertension (OR 4.01, CI 1.05–15.36, *I*^2^ = 77%), heart disease (OR 2.53, CI 1.01–6.33), diabetes (OR 2.31, CI 1.15–4.65), dyslipidemia (OR 2.13, CI 1.52–3.00), smoking (OR 1.77, CI 1.21–2.59), and male sex (OR 1.71, CI 1.24–2.36). Low levels of nonsignificant heterogeneity were recorded for the analysis of disease severity (*I*^2^ = 39%, *P* = 0.15), dyslipidemia (*I*^2^ = 0%, *P* = 0.42), smoking (*I*^2^ = 0%, 0.74), and male sex (*I*^2^ = 10%, *P* = 0.35). However, considerable and significant heterogeneity was recorded for hypertension (*I*^2^ = 39%, *P* = 0.004), diabetes (*I*^2^ = 63%, *P* = 0.03), and heart disease (*I*^2^ = 76%, *P* = 0.002). Malignancy, chronic kidney disease (CKD), and a history of neurological diseases were not significant risk factors (OR 1.23, CI 0.41–3.66; OR 1.24, CI 0.55–2.79; OR 2.13, CI 0.66–6.88, respectively). Figure 5 shows a forest plot of the pooled estimate of the significant risk factors for neurological association among COVID-19 patients.

## Discussion

### Summary of the Main Results

The current review included 4401 COVID-19 patients from six observational studies. Of these, 8.24% developed neurological complications, including delirium (84.3%), myalgia (44.8%), headache (37.7%), encephalopathy (31.8%), dizziness (29.7%), dysgeusia (15.9%), anosmia (11.45), acute ischemic stroke (4.6%), cerebrovascular disease (1.78%), and intracerebral hemorrhage (0.5%). Movement disorders, motor and sensory deficits, ataxia, and seizures, were uncommon and accounted for 0.2–1.4% of cases. In COVID-19 patients, the severity of COVID-19 disease, along with underlying comorbidity, but particularly hypertension, increased the risk for neurological complications by approximately fourfold (OR 4.30, CI 2.54–7.29 and OR 4.01, CI 1.05–15.36 respectively). Heart disease, diabetes, and dyslipidemia increased the risk by approximately twofold (OR 2.53, CI 1.01–6.33; OR 2.31, CI 1.15–4.65; and OR 2.13, CI 1.52–3.00, respectively). Other recorded risk factors were old age (MD 18.35, CI 9.39–27.32), male sex (OR 1.71, CI 1.24–2.36) and smoking (OR 1.77, CI 1.21–2.59). Malignancy, CKD, and a history of neurological diseases were not significant risk factors (OR 1.23, CI 0.41–3.66; OR 1.24, CI 0.55–2.79; OR 2.13, CI 0.66–6.88, respectively).

### Quality of Evidence

Overall, we downgraded the quality of evidence by one level for all outcome measures due to the observational design of the included studies. In Table 2, we determined the mean difference (MD) among participants of included articles regarding different aspects, including mean age and male sex. The heterogeneity in the mean age of the included articles showed to be significant (*p* < 0.0001). This revealed that difference of mean age in the included studies is large. Low to moderate insignificant heterogeneity was recorded for the pooled estimate for male sex, the severity of COVID-19, smoking, dyslipidemia, CKD, and malignancy (*I*^2^ = 10%, 39, 0, 0, 57, and 60%, *P* > 0.05 respectively); thus, the quality of evidence for the pooled estimate of these outcomes was judged to be moderate. However, we judged the quality of evidence to be low for the pooled estimate of the following outcomes: mean age, hypertension, diabetes, heart disease and a history of neurological diseases. We downgraded the evidence by one more level due to the considerable significant heterogeneity recorded in these analysis (*I*^2^ = 93%, 77, 63, 76, and 69%, *P* < 0.05 respectively); this heterogeneity could be explained by differences among participants in the background variables and methods used for neurological diagnosis and case definition (Table 2).

**Table 2:** Quality of evidence relating to outcome measures

Outcome	Number of participants	OR/MD	CI	Heterogeneity	Quality of evidence
Mean age	2318	18.35	9.39–27.32	*I*^2^ = 93%, *P* < 0.0001	Low
Male sex	4401	1.71	1.24–2.36	*I*^2^ = 10%, *P* = 0.35	Moderate
Smoking	2099	1.77	1.21–2.59	*I*^2^ = 0%, *P* = 0.74	Moderate
Severity	4401	4.30	2.54–7.29	*I*^2^ = 39%, *P* = 0.15	Moderate
Hypertension	2671	4.01	1.05–15.36	*I*^2^ = 77%, *P* = 0.0004	Low
Diabetes	2811	2.31	1.15–4.65	*I*^2^ = 63%, *P* = 0.03	Low
Dyslipidemia	2425	2.13	1.52–3.00	*I*^2^ = 0%, *P* = 0.42	Moderate
HD	2811	2.53	1.01–6.33	*I*^2^ = 76%, *P* = 0.002	Low
CKD	2592	1.24	0.55–2.79	*I*^2^ = 57%, *P* = 0.07	Moderate
Malignancy	895	1.23	0.41–3.66	*I*^2^ = 60%, *P* = 0.06	Moderate
History of neurological disease	868	2.13	0.66–6.88	*I*^2^ = 69%, *P* = 0.04	Low

*CKD* chronic kidney disease, *HD* heart disease, *MD* mean difference, *OR* odds ratio

### Overall Completeness and Applicability of Evidence

All of the studies included in this review prespecified and determined neurological complications in COVID-19 patients as the primary outcome measure. Furthermore, most studies investigated and reported the following risk factors: male sex, the severity of COVID-19, hypertension, diabetes, heart disease, malignancy, and CKD. Mean age, dyslipidemia, and smoking were reported by two studies only.

### Potential Bias Encountered During the Review Process

For the current review, two authors independently and systematically screened major databases. In addition, the authors independently extracted and data and performed analysis. The authors solved any disagreement or variation in judgment by discussion. Therefore, it is unlikely that this approach could have introduced bias in the review.

### Agreements and Disagreements with Other Studies and Reviews

#### Frequency of Neurological Complications in COVID-19 Patients

In this review, we recorded neurological complications in 8.24% of patients with COVID-19, including acute ischemic stroke (4.6%), intracerebral hemorrhage (0.5%), cerebrovascular disease (1.78%), delirium (84.3%), myalgia (44.8%), headache (37.7%), encephalopathy (31.8%), dizziness (29.7%), dysgeusia (15.9%), and anosmia (11.45). Motor and sensory disorders, ataxia, and seizures were rare (accounting for 0.2–1.4% of cases). Recent studies have reported that some COVID-19 patients develop neurological diseases in addition to the more common upper and lower respiratory symptoms, including viral encephalitis, meningoencephalitis, ischemic stroke, and hemorrhagic stroke [8–10, 25, 26]. Furthermore, Mao et al. [8] investigated hospitalized COVID-19 patients and found that 36.4% had nervous system manifestations in the CNS (24.8%), PNS (8.9%), and skeletal muscle injury (10.7%). These authors also reported that the most common CNS manifestations were dizziness (16.8%) and headache (13.1%). The most frequently reported PNS symptoms were taste (5.6%) and the impairment of smell (5.1%).

Similarly, Moriguchi et al. [9] and Xiang et al. [10] reported two cases of encephalitis in COVID-19 patients. Rothstein et al. [27] recorded ischemic stroke and intracranial hemorrhage in 2.4 and 0.9% of hospitalized COVID-19 patients, respectively. Furthermore, Guillain–Barre syndrome has been identified in COVID-19 patients in many case reports [28–30].

However, Ghannam et al. [31] recorded higher figures for the pooled estimate of neurological complications in COVID-19 patients, including cerebrovascular insults (48.8%), neuromuscular disorders (28%), and encephalitis (23%). These high figures could be explained by the low number of included participants and the different methods used for case definition.

#### Possible Mechanisms Underlying Neurological Complications

Inflammation of the CNS in COVID-19 patients could be caused by nonspecific complications of systemic disorder [6]. An alternative proposal theorized that inflammation of the CNS could be caused by viral infection via the axonal transport of the virus via the peripheral nerves into the brain [7]. Brain injury and neurological symptoms might occur because of the invasion of neural cell membranes by ACE2. The spike protein of the coronavirus is known to bind to neuronal ACE2 receptors in the brain and attaches to target cells. Then, the spike protein is activated by serine protease TMPRSS2, thus allowing the virus to enter neurons [32]. In addition, endothelial cells in the cerebral blood vessels might be attacked by the virus through ACE2 receptors, thereby disrupting the blood–brain barriers and promoting invasion of the brain tissue and neurons [33, 34].

#### Risk Factors for the Development of Neurological Complications

In this review, we investigated factors associated with the development of neurological complications in COVID-19 patients. The severity of COVID-19 disease and underlying comorbidity (mainly hypertension) increased the probability of the risk by approximately fourfold (OR 4.30, CI 2.54–7.29 and OR 4.01, CI 1.05–15.36, respectively). Heart disease, diabetes, and dyslipidemia increased the risk by approximately twofold (OR 2.53, CI 1.01–6.33; OR 2.31, CI 1.15–4.65; and OR 2.13, CI 1.52–3.00, respectively). Other recorded risk factors were old age (MD 18.35, CI 9.39–27.32), male sex (OR 1.71, CI 1.24–2.36), and smoking (OR 1.77, CI 1.21–2.59). On the other hand, malignancy, CKD, and a history of neurological disease were not significant risk factors (OR 1.23, CI 0.41–3.66; OR 1.24, CI 0.55–2.79; OR 2.13, CI 0.66–6.88, respectively). These findings were consistent with those of Mao et al. [8] who found that patients with severe COVID-19, along with those with underlying disorders, especially hypertension, developed significant higher percentages of acute cerebrovascular disease (mainly ischemic stroke) (45.5 vs. 30.2%, *P* = 0.02 and 5.7 vs. 0.8%, *P* = 0.03 respectively). Similarly, Rothstein et al. [27] found that ischemic stroke was more prevalent in hypertensive, diabetic, and elderly patients with COVID-19. Bekelis et al. [35] reported that COVID-19 patients who develop ischemic stroke were associated with a higher case-fatality rate (OR 10.50, CI 3.54–31.18) and had an increased risk for discharge to rehabilitation (OR 2.45, CI 0.81–1.25). Similarly, Ojo et al. [36] reported similar risk factors for both severe COVID-19 and ischemic stroke. Another study also found that COVID-19 patients who developed ischemic stroke had at least one stroke-associated comorbidity [37].

Along the same lines, Ghannam et al. [31] found that the mean age of COVID-19 patients who developed neurological complications was 62.3 years; moreover, 62.2% of these were males.

Similarly, Kennedy et al. [38] reported that the pooled incidence of intracranial hemorrhage among patients with COVID-19 was 0.7% (CI 0.5–0.9); most patients were elderly men (66%) diagnosed with hypertension (54%).

Similarly, a prospective study identified risk factors associated with the development of delirium among COVID-19 patients including an older age (adjusted relative risk [aRR] 1.51, CI 1.17–1.95), living in a nursing home (aRR 1.23, CI 0.98–1.55), and both vision and hearing impairment (aRR 1.98, CI 1.54–2.54 and aRR 1.10, CI 0.78–1.55, respectively). However, in contrast with our findings, a past history of neurological disease, such as stroke, Parkinson disease, and previous use of psychoactive medication, were all identified as significant risk factors (aRR 1.47, CI 1.15–1.88; aRR 1.88, CI 1.30–2.58; and aRR 1.42, CI 1.11–1.81, respectively) [39].

Similar risk factors for neurological complications in COVID-19 patients were reported by Pun et al. [40], including an older age (OR 1.13, CI 1.03–1.25), a higher Simplified Acute Physiology Score (SAPS) (OR 1.17, CI 1.07–1.29), smoking or alcohol abuse (OR 1.37, CI 1.13–1.67), and disease severity, such as invasive mechanical ventilation (OR 1.48, CI 1.17–1.87). These authors also identified the following risk factors: vasopressors (OR 1.25, CI 1.10–1.43), the use of restraints (OR 1.32, CI 1.16–1.50), antipsychotics (OR 1.59, CI 1.36–1.85, *P* < 0.0001), sedative benzodiazepine infusions (OR 1.59, CI 1.33–1.91), and continuous opioid infusions (OR 1.39, CI 1.21–1.60). Family visitation, however, was associated with a 27% lower risk of delirium (OR 0.73, CI 0.63–0.84, *P* < 0.0001).

In addition, previous studies reported that patients with COVID-19 who were admitted with neurological diseases, including stroke, had significantly higher rates for admission into the intensive care unit [39], in-hospital mortality, incident delirium [41], and discharge to rehabilitation [35].

## Conclusion

In this review, we identified several significant factors associated with the development of neurological complications in COVID-19 patients, including old age, male sex, smoking, severity of the disease and underlying comorbidities including hypertension, heart disease, diabetes, dyslipidemia. These complications are relatively uncommon. However, during the COVID-19 pandemic, differential diagnosis of the disease should be suspected when confronted with patients with neurological manifestations to avoid delayed diagnosis or misdiagnosis. Early identification of at-risk patients, particularly experiencing comorbid conditions could be important if we are to improve their outcome and prevent further transmission.

## Data Availability

The data that support the findings of this study are available from the corresponding author, upon reasonable request.

## Abbreviations

ACE2: Angiotensin converting enzyme-2
aRR: Adjusted relative risk
CI: Confidence interval
CKD: Chronic kidney disease
CNS: Central nervous system
COVID-19: Coronavirus disease 2019
HD: Heart disease
MD: Mean difference
MRI: Magnetic resonance image
OR: Odds ratio
PNS: Peripheral nervous system
SAPS: Simplified Acute Physiology Score
SARS-CoV-2: Severe acute respiratory syndrome coronavirus 2
TMPRSS2: Transmembrane protease serine 2
WHO: World Health Organization
